# Recombinant protein expression in microbial systems

**DOI:** 10.3389/fmicb.2014.00341

**Published:** 2014-07-08

**Authors:** Germán L. Rosano, Eduardo A. Ceccarelli

**Affiliations:** Instituto de Biología Molecular y Celular de Rosario, Consejo Nacional de Investigaciones Científicas y Técnicas, Facultad de Ciencias Bioquímicas y Farmacéuticas, Universidad Nacional de RosarioRosario, Argentina

**Keywords:** recombinant proteins, microorganism, inclusion bodies, fusion tags, *Escherichia coli*, yeast, filamentous fungi, microalgae

## Introduction

The emergence of recombinant DNA technology during the early 70's set a revolution in molecular biology. This set of techniques was strengthened even further later on with the introduction of the polymerase chain reaction and allowed scientists to explore and understand essential life processes in an easy and straightforward way. It also marked the birth of the modern biotech industry. At that time, it was shown that eukaryotic DNA could be propagated in *Escherichia coli* (Morrow et al., 1974) and functional products could be synthesized from heterologous genes cloned in bacterial plasmids (Ratzkin and Carbon, 1977; Vapnek et al., 1977). After these successful cases, it was soon realized that the potential applications of these techniques were almost limitless. In fact, US patent 4,237,224 granted to Cohen and Boyer (1980) claimed to commercial ownership of the methodology for cloning virtually all possible DNAs in all possible vectors. While cloning any gene in any given vector is feasible, obtaining a functional product from its expression is not that simple.

In this series of articles, the authors describe the methods and technologies available for producing recombinant proteins in different microbes. They also introduce and discuss recent advances that attempt to tackle common pitfalls in the process. Taken together, this E-book will be of great importance for those entering the field as well as for experienced researchers that are looking for an update in the state of the art.

Before proceeding any further, it is necessary to clarify an important aspect of this topic. In biology, the universal accepted definition of “expression” is “production of an observable phenotype by a gene—usually by directing the synthesis of a protein” (Alberts et al., 2002). By this definition, the term “gene expression” is correct while “protein expression” is basically lab jargon. We do think that correct usage of scientific language is of great importance, yet in this particular case, the usage of “protein expression” in the scientific community is so pervasive that readers will immediately understand what we are talking about. So, considering that “protein expression” found its way into journal names, book names and high-impact reviews (Sørensen and Mortensen, 2005) and research papers (Ghaemmaghami et al., 2003) (>1800 citations in Scopus) we and other authors have used it interchangeably with more correct terms like protein production or protein synthesis.

## Current status in recombinant protein expression in microbial systems

Without a doubt, *E. coli* is the most widely used host for heterologous gene expression. It has been used for this purpose for more than 40 years, so there is much accumulated knowledge about its advantages and disadvantages as an expression platform. Rosano and Ceccarelli review the tools at hand (expression vectors, strains, media composition, etc.) when using *E. coli* as a host (Rosano and Ceccarelli, 2014). Different approaches for solving common problems, such as inclusion body (IB) formation or low yield, are also presented.

Other authors delve a little deeper into these issues. Costa and coauthors give a thorough description of different fusion tags that can be appended to the target protein in order to increase its solubility and/or ease its purification from the cellular milieu (Costa et al., 2014). Along this line, Correa and coauthors present a very promising approach to straightforwardly assess the solubility of a recombinant protein by cloning the corresponding gene in 12 different expression vectors in parallel (Correa et al., 2014). Even though IB formation is mainly regarded as a nuance in the production of recombinant proteins, Ramon and coworkers make the case that this is not always true and focus on the positive side of IBs, highlighting the advantages of producing recombinant proteins as IBs for basic and applied research (Ramon et al., 2014). No or low yield can be the result of codon bias and Elena and coauthors look into one of the strategies used for solving this problem, the expression of codon optimized genes (Elena et al., 2014). Of great importance is their account of the application of this technology in the industrial setting. Not always the desired product is a protein, sometimes metabolites and fine chemicals are the goal. The review of Ceccoli and coauthors details with numerous examples how *E. coli* and other microorganisms can be turned into biocatalysts by strain engineering (Ceccoli et al., 2014). High-value products can thus be obtained from pure recombinant enzyme or from whole-cell systems, turning the host into an exquisitely designed and environmentally friendly chemical factory.

## Advances in new technologies

Microbes other than *E. coli* can be used for heterologous protein production. The impact of yeasts on the biotech industry is paramount, as 20% of biopharmaceutic proteins are synthesized in yeasts. But still, they are not the first-choice microorganism for recombinant protein production. In her Perspective article, Bill arguments that the yeasts *Saccharomyces cerevisiae* and *Pichia pastoris* should be considered alongside *E. coli* in any project in need of a recombinant protein (Bill, 2014). Filamentous fungi are excellent protein secretors, a property that makes them ideal as a host at an industrial scale. Nevalainen and Peterson present an authoritative review describing the body of research carried out into cellular mechanisms of fungi that will ultimately lead to the optimization of the system (Nevalainen and Peterson, 2014). Specht and Mayfield explain the usefulness of microalgae as expression systems, focusing on the production of recombinant vaccines (Specht and Mayfield, 2014). The quest for edible vaccines is an active area of research and microalgae will definitely play a major role as they possess greater advantages over plants (which can also be used for this purpose) in terms of cost, safety, and logistics.

## Afterword

After getting into all the articles in this E-book, it should be clear to the reader that progress in the field of recombinant protein expression in microbial systems shows no sign of deceleration. Their use and importance in research and in industry cannot be disputed. Their establishment in the bio(techno)logy toolkit was due to the ongoing efforts of researchers that continuously optimize well-known systems such as *E. coli* and those who break new ground in the use of alternative microorganisms. We want to take this opportunity to thank all the experts of this series for their excellent contributions and the reviewers for their insightful comments and remarks.

### Conflict of interest statement

The authors declare that the research was conducted in the absence of any commercial or financial relationships that could be construed as a potential conflict of interest.
